# Comparative transcriptome analysis highlights the crucial roles of photosynthetic system in drought stress adaptation in upland rice

**DOI:** 10.1038/srep19349

**Published:** 2016-01-18

**Authors:** Zheng-Feng Zhang, Yuan-Yuan Li, Ben-Ze Xiao

**Affiliations:** 1 Hubei Key Laboratory of Genetic Regulation and Integrative Biology, School of Life Sciences, Central China Normal University, Wuhan 430079, People’s Republic of China; 2 College of Plant Science and Technology, Huazhong Agricultural University, Wuhan 430070, People’s Republic of China

## Abstract

Drought stress is one of the major adverse environmental factors reducing plant growth. With the aim to elucidate the underlying molecular basis of rice response to drought stress, comparative transcriptome analysis was conducted between drought susceptible rice cultivar Zhenshan97 and tolerant cultivar IRAT109 at the seedling stage. 436 genes showed differential expression and mainly enriched in the Gene Ontology (GO) terms of stress defence. A large number of variations exist between these two genotypes including 2564 high-quality insertion and deletions (INDELs) and 70,264 single nucleotide polymorphism (SNPs). 1041 orthologous gene pairs show the ratio of nonsynonymous nucleotide substitution rate to synonymous nucleotide substitutions rate (Ka/Ks) larger than 1.5, indicating the rapid adaptation to different environments during domestication. GO and Kyoto Encyclopedia of Genes and Genomes (KEGG) enrichment analysis of positive selection genes suggested that photosynthesis represents the most significant category. The collocation of positively selected genes with the QTLs of photosynthesis and the different photosynthesis performance of these two cultivars further illuminate the crucial function of photosynthesis in rice adaptation to drought stress. Our results also provide fruitful functional markers and candidate genes for future genetic research and improvement of drought tolerance in rice.

Drought stress is one of the major adverse environmental factors comprising plant growth and productivity. It negatively affects various plant processes including growth, physiology, metabolism and transcription^1^,^2^. Plants evolved mechanisms to water deficit during the long period of domestication. Paddy rice Zhengshan97 and upland rice IRAT109 are such various resources characterized as drought sensitive and drought tolerant, respectively^3^. These two rice cultivars show distinct characters to water stress but are identical in most of the morphological and developmental traits. Multi-scale research experiments have been carried out using paddy and upland rice as bio-materials, for detection of quantitative trait loci (QTLs) related to drought stress^4^,^5^,^6^,^7^, exploration of differentially expressed genes (DEGs) using microarray or suppression subtractive hybridization (SSH) technique^8^,^9^,^10^, phenotypic and physiological analysis^4^,^11^ as well as genome-wide comparative analysis, which revealed genetic mechanisms underlying upland adaptation in rice^12^.

During the divergence of the upland and lowland rice, the former must have acquired a range of adaptive mechanisms to cope with the harsh features of upland environment especially drought^4^,^5^,^13^. Selection related to domestication has modified a number of traits that now distinguish the modern rice accessions^12^,^14^,^15^,^16^. Signature of selection during domestication have prominent role at loci linked to the selection target traits^17^. Ka/Ks ratio is a key indicator telling us the way the genes have evolved or been domesticated. The Ka/Ks much greater than 1 is the strong evidence that positive selection has acted to change the protein^18^. Distinctive phenotype responding to severe environments can be generated under the positive selection on some key genome loci^19^.

Comparing transcriptomes of various genotypes of specific species is useful for exploring genes under selection and elucidating the role of various biological pathways and mechanisms for imparting stress tolerance to adverse environments^20^,^21^. Although fundamental researches have provided significant insights into the physiological and molecular responses of plants to water deficit, but the divergence in transcriptome of rice genotypes with contrasting phenotypes remains unexplored. Techniques such as next-generation sequencing offer a unique opportunity to scan the transcriptomes of different genotypes. In the present study, the comparative transcriptomes of drought-tolerant rice cultivar IRAT109 and sensitive rice cultivar Zhenshan97 were analyzed comprehensively to provide insights into sequence variation, expression divergence, domestication selection of genes between drought-tolerant and sensitive rice genotypes under drought conditions. We highlight the crucial role of photosynthetic system during the domestication of these two cultivars, which experience strong positive selection during the domestication of rice cultivars to upland water deficit environments. Moreover, this study provides functional gene markers to promote future relevant research, and molecular breeding and genetic engineering projects for enhancing drought tolerance in rice.

## Results

### Transcripts assembly

To get deeper insight into the drought stress responses of upland drought-resistant (IRAT109) and lowland drought-sensitive (Zhenshan 97) rice cultivars, a genome-wide transcriptional analysis was performed by using Illumina Hiseq2500 platform. Approximately 30.6 million 100bp paired-end clean reads pairs for per library were generated after adapter trimming and filtering low-quality reads. On average, about 53.7 and 51.3 million reads were uniquely mapped to the reference genome with tophat default parameters criteria in Zhenshan97 and IRAT109 respectively. Among of which, 83.2% and 85.4% of reads were properly paired mapped respectively for each sample. Transcript prediction from the present samples were initially performed separately, yet generated a transcript set with a high degree of overlap. Merging of the predicted transcripts sets resulted in 103490 transcripts with 34832 novel transcripts which were not annotated in rice gene model (ftp://ussd-ftp.illumina.com/Oryza_sativa_japonica/Ensembl/MSU6/) (Supplemental Table 1). The novel transcripts indicate the alternative splicing events increase the transcripts diversity.

### Differential expressed genes and Gene Ontology analysis

**Figure 1:**
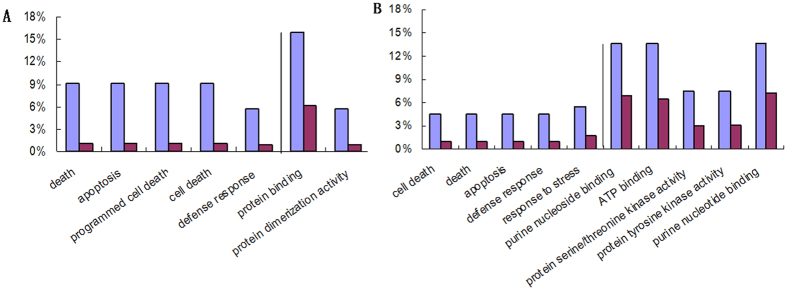
GO enrichment of DEGs between the upland and paddy rice. (**A**) Significant GO terms of genes up-regulated or specific expressed in Zhenshan97. (**B**) Significant GO terms of genes up-regulated or specific expressed in IRAT109. The blue bar indicate the percent of query genes in a specific GO terms to the total query genes and the purple bar indicate that for the background. The left and right of the vertical line indicate the biological process and molecular function GO terms, respectively.

Comparing upland and lowland rice cultivars under drought conditions resulted in a total of 436 genes differentially expressed genes, which were classified into 8 categories according to the log10 (FPKM + 1) value of gene expression by K-means (Supplemental Fig. 1). Among 436 genes, 79 (18%) were new ones without equivalents in the current annotated genes. The rest of 357 genes included 115 up or specially regulated in Zhenshan97 and 242 of that in IRAT109. To further look into the functional categories of genes differentially expressed between upland and lowland rice, Gene Ontology analysis was implemented by searching the upland-up and lowland-up regulated genes against the AgriGO database of Oryza sativa Japonica (Fig. 1). The up and specific regulated genes in Zhenshan97 are enriched in 7 most significant GO terms (P-value < 0.01) including 5 biological process GO terms as include death, cell death, programmed cell death, apoptosis and defense response and 2 significant molecular function GO terms of protein binding and protein dimerization activity. The up and specific regulated genes in IRAT109 are enriched in 10 most significant GO terms (P-value < 0.01) covering death, cell death, programmed cell death, apoptosis, response to stimulus, response to stress and defense response. The function description and expressed value of genes included in the above significant GO terms were listed (Supplemental Table 2). The genes specific or upregulated in Zhenshan97 are mainly enriched in significant GO terms mainly encode disease resistance proteins, transposon proteins, receptor-like protein kinase precursor and so on. The genes specific or up regulated in IRAT109 enriched in significant GO terms mainly include serine/threonine-protein kinase and ATP-binding proteins.

**Figure 2:**
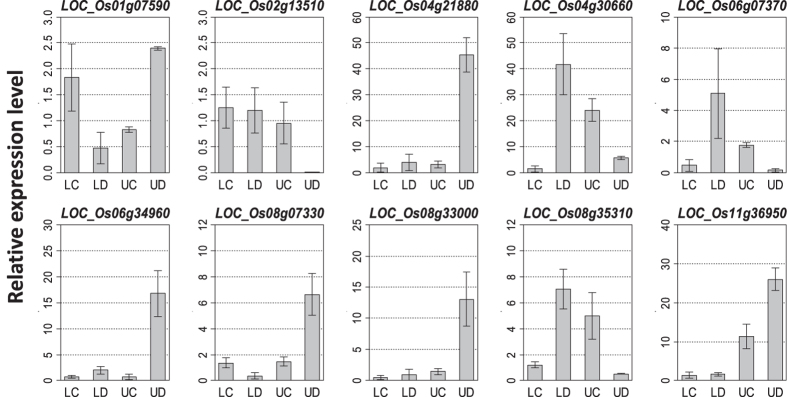
Validation of 10 DEGs by qRT-PCR. qRT-PCR values were calculated as means from all relevant varieties for Lowland rice under Control (LC) and Drought stress (LD) as well as for Upland rice under Control (UC) and Drought stress (UD). Error bars indicate the standard deviation. The X-axis indicates the rice materials and growth condition. Y axis indicates the relative expression level.

To validate the RNA sequencing gene expression results, quantitative real-time reverse transcription-PCR (qRT-PCR) was used to assess the expression levels for 10 randomly selected genes in five lowland rice varieties and three upland rice varieties (Plant materials and methods). Because gene express analysis from RNA sequencing were not performed for control condition, only the results analyzed from drought stress were compared between RNA sequencing and qRT-PCR. By comparing the relative expression levels for the 10 genes under drought conditions between these two methods, consistent expression trends were observed for all the selected genes (Fig. 2 and Supplemental Table 2). For genes Os01g07590, Os04g21880, Os06g34960, Os08g07330, Os08g33000, Os11g36950, their expressions were very low even hardly to be detected in lowland rice cultivars whereas show comparative high expression amount in upland rice as revealed by RNA sequenceing. Consistently, the qRT-PCR produced results that these gene expression ratios of upland to lowland rice under drought stress are 5.07, 11.86, 8.42, 17.23, 13.91 and 16.31 respectively. In contrast, genes Os02g13510, Os04g30660, Os06g07370 and Os08g35310 show profoundly decreased expressions under upland rice compared to the lowland rice as detected by RNA sequencing. The qRT-PCR results revealed these four gene expression ratios of lowland to upland rice under drought stress are 144.33, 6.61, 33.06, 14.11 respectively, which indicated a good agreement between both approaches (Fig. 2). It is worth mentioning that except LOC_Os01g07590 and LOC_Os02g13510, the rest of the eight genes show expression FPKM value as 0 in upland or lowland rice detected by RNA sequencing (Supplemental Table 2), whereas revealed a relatively low expression value as indicated by qRT-PCR (Fig. 2). This is probably because a much higher coverage is necessary in RNA sequencing for detecting the expression for those mRNAs with extremely low amount.

### Identification, experimental validation and distribution of SNPs and INDELs along chromosomes

**Figure 3:**
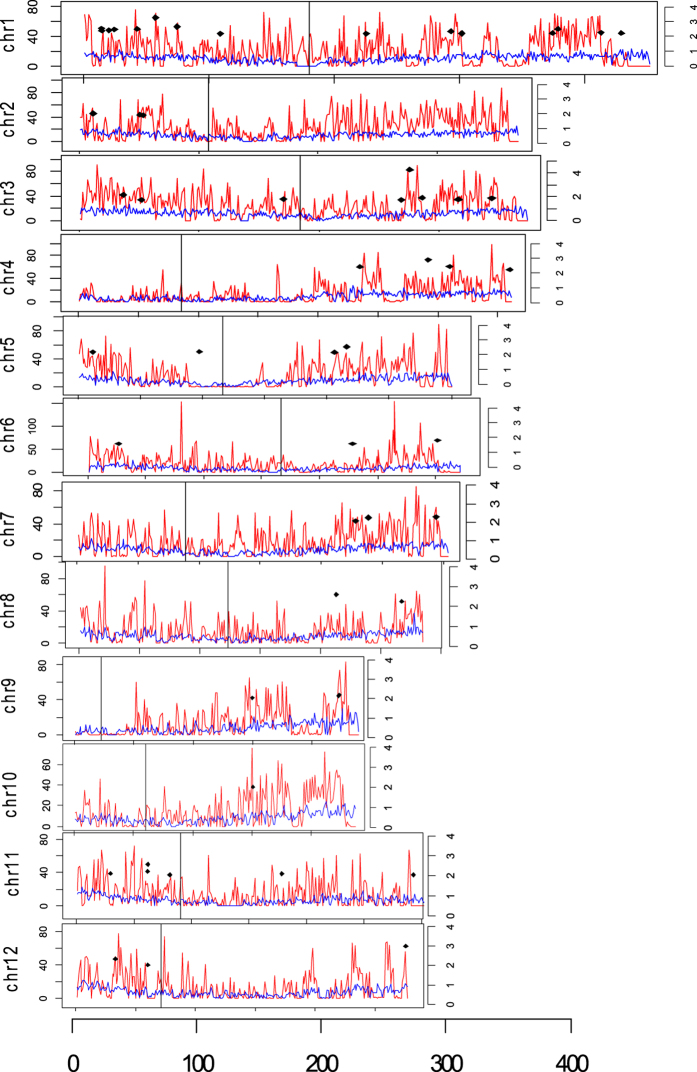
The distribution of sequence variations (SNPs and INDELs) and genes along the chromosomes. The left Y-axis shows the number of sequence variations and genes. The right Y-axis indicates the Ka/Ks value of genes. The X-axis shows the position on the chromosomes with the scale as 100kb. The red and blue lines indicate the numbers of sequence variations and genes in 100kb slide windows, respectively. The black point in the graph represents the genes with Ka/Ks value bigger than 2. The detailed information about the ids and function annotation of these genes were summarized in supplementary table 3. The black vertical lines shows the positions of centromere.

The variations calling by Samtool package generates total 166,538 raw variations between samples and the reference. The 110,826 variations were remained after removing those with read depth (DP) smaller than 5, phred-scaled quality (an overall quality score for the assertion made in alternate non-reference alleles) smaller than 30 and mapping quality (Root-mean-square mapping quality of covering reads) smaller than 20. As the variations output directly from the SAMtools mpileup scripts show the variations between the reference genome (cultivar Nipponbare of *Oryza sativa ssp. japonica*) and each investigated genotype (Zhenshan97 or IRAT109) separately, whereas the aim to the present study is to search the variations between lowland rice Zhenshan97 and upland rice IRAT109. To distinguish the variations between the two investigated genotypes apart from variations between reference and genotypes, an in-house bash script was used following variations calling with SAMtools mpileup. Finally we screened 77,647 variation sites between the two studied genotypes and 72,840 of which are homologous sites in both genotypes including 70,264 SNPs and 2576 INDELs (Table 1). To further look into if there is some hot spots with high-density of functional SNPs and INDELs in the genome, we calculated the SNPs, INDELs and gene density along the chromosomes in 100kb sliding windows (Fig. 3). We observed that the SNPs and INDELs density was generally low around centromeres and telomeres, particularly around the centromeres of chromosomes 4, 5, 9 and the proximal end of long arm of chromosome 9. Consistently, the gene density of these regions are also relatively low.

**Figure 4:**
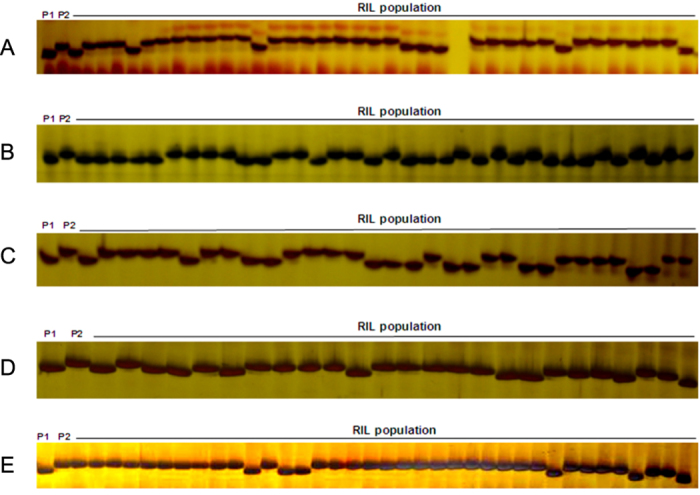
The representative INDELs shown through PAGE and silver staining. These 2bp-INDELs A-E represents INDEL1_146103_CT, INDEL3_7749032_GT, INDEL7_6809650_AG, INDEL8_14321370_AA and INDEL9_14716537_AG, respectively. For the nomenclature of these INDELs, number after the word INDEL indicates the chromosome, the number after the first underline indicates the position on the corresponding chromosome, and the two nucleotides after the second underline is the INDEL. P1 and P2 on the top of each gel track represents the Zhenshan97 and IRAT109, and the bands after P1 and P2 represent individuals from the RIL population which was originated from Zhenshan97 and IRAT109.

To validate the reliability of these sequences variations with experiments, 200 2bp-INDELs were selected randomly for experimental validation with the recombinant inbred lines (RILs) generated with IRAT109 and Zhenshan97. 189 of them were validated through Polymerase Chain Reaction (PCR) amplification and PAGE (polyacrylamide gels electrophoresis). The representative INDELs were shown in Fig. 4. Our results suggest that the short INDELs like 2 nucleotides polymorphism can be clearly distinguished by traditional gels analysis if the PCR products were designed in length of 150-200bp and separated on the higher resolution PAGE.

### The Ka/Ks Calculation for orthologs between IRAT109 and Zhenshan97

**Figure 5:**
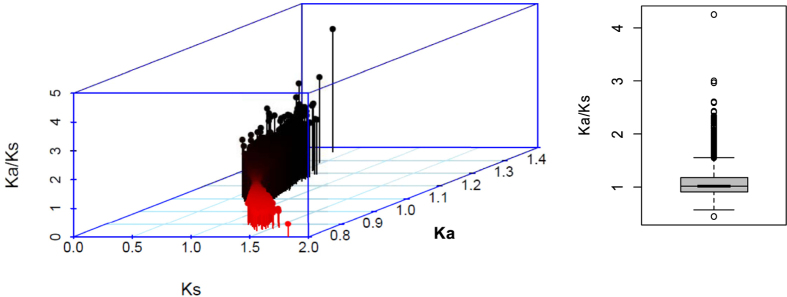
The synonymous, nonsynonymous mutatation rates and Ka/Ks value of orthologs between the upland and lowland rice accessions in the present study. The 3D scatter plot at the left shows the Ks at X-axis, Ka at Y-axis and Ka/Ks at the Z-axis. The right box plot displays the range of Ka/Ks values and with the mean of about 1.

To identify genes undergoing positive selection during the domestication of the upland and lowland rice cultivars, the ratios of non-synonymous to synonymous mutations for orthologs between the two genotypes were calculated. Using the bidirectional Blast between the transcripts of the two accessions and the annotated proteins sequences in gene model as references, 12626 orthologous pairs were obtained for further Ka/Ks calculation. Except 4 genes of which showed Ka/Ks of zero, the rest of the orthologs show a range of Ka/Ks values between 0.44 and 4.25, with an average of 1.078, indicating that most of the genes were subjected to the neutral selection (Fig. 5). 1041 of orthologous pairs shows Ka/Ks values over 1.5 and 54 of which with Ka/Ks values bigger than 2 (Supplemental Table 3). These genes could be considered as genes undergoing strong positive selection during domestication to different growth environments.

### Functions of genes with Ka/Ks values greater than 1.5

**Figure 6:**
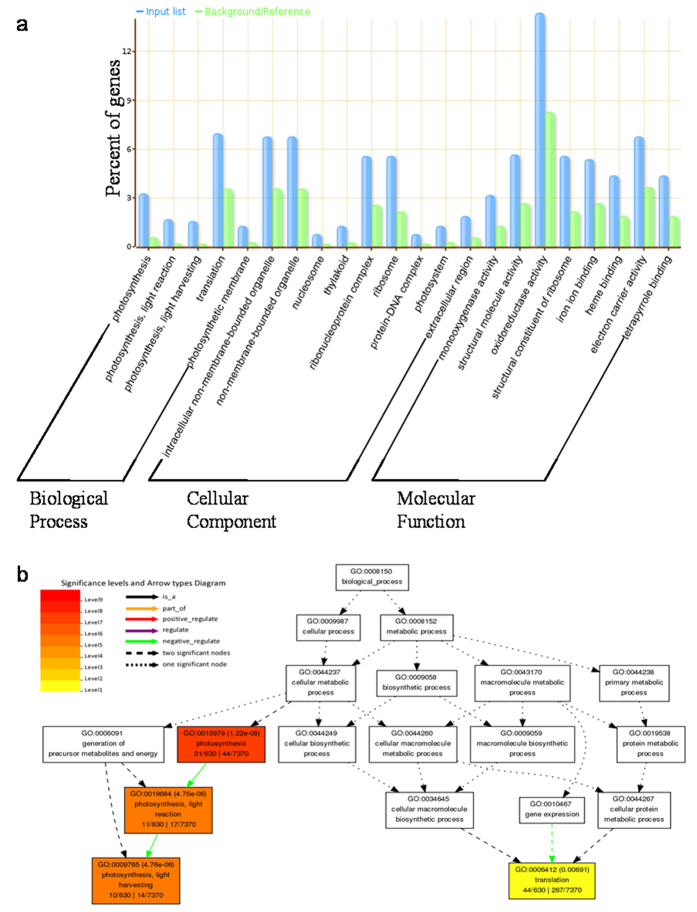
Go enrichment analysis for genes with Ka/Ks > 1.5. (**a**) The significant biological process, cell compartment and molecular function GO terms are shown from left to right. Photosynthesis represents the most significant GO terms in biological process. (**b**) The categories of biological processes GO terms are shown as a diagram. The darker the box color, the significant level is higher.

**Figure 7:**
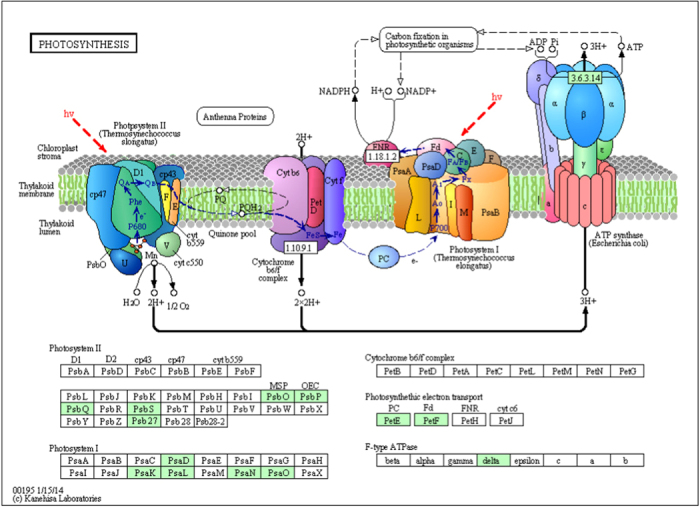
The KEGG photosynthesis pathway including many genes with Ka/Ks > 1.5. The KEGG photosynthesis pathway map can be found online at http://www.kegg.jp/pathway/map00195. The light green boxes indicate the proteins encoded by the genes with Ka/Ks value bigger than 1.5. The detail annotation of these genes were summarized in Supplemental Table 4.

1041 genes with the Ka/Ks value more than 1.5 were subjected to KEGG pathway construction analysis. With the aim to focus on the function in which positively selected genes involved and prevent the affect of genes expressed in leaves,these genes were also under GO test with 12626 orthologous genes as customized reference, instead of the whole annotated rice genes. 40 significant GO terms were retrieved with P-value < 0.01 and considerable amount of genes were enriched in photosynthesis, gene expression and oxidoreductase activity GO categories (Additional file 2). 22 most significant GO terms were shown (Fig. 6a) and the significant biological process GO terms were separately displayed (Fig. 6b). Photosystem related GO terms were emphasized as their high level of significance. KEGG pathway construction analysis revealed that genes were involved in photosynthesis and oxidative phosphorylation pathways (Fig. 7, Additional file 1 Supplemental Table 4). Since a large number of QTLs associated with drought response have been reported to be associated with drought tolerance, therefore we were curious to inspect whether these positively selected genes collocates with photosynthesis related QTLs which are also involved in drought tolerance. Interesting, 9 and 8 genes with Ka/Ks value bigger than 1.5 were collocated with photosynthesis-related QTLs^22^,^23^ and leaf phenotypes related QTLs^7^, respectively (Table 2). A cluster of QTLs^22^ for photosynthesis parameters in rice leaves were located near marker RM410 (the interval from 57.3 cM to 68.4 cM on chromosome 9) and four positively selected genes *viz.* Os09g28310, Os09g28354, Os09g29130 and Os09g29490 were collocated closely with four photosynthesis parameters QTLs near RM410 and encoding proteins with bZIP transcription factor, heat shock factor, ZF-HD protein dimerization and peroxidase, respectively. Three positive selected genes *viz.* Os01g39780, Os01g64960 and Os04g59440 encode putative photosynthesis-associated functions such as located in plastid and chlorophyll a-b binding are also mapped near the photosynthesis parameters QTLs (Table 2).

### Photosynthesis

**Figure 8:**
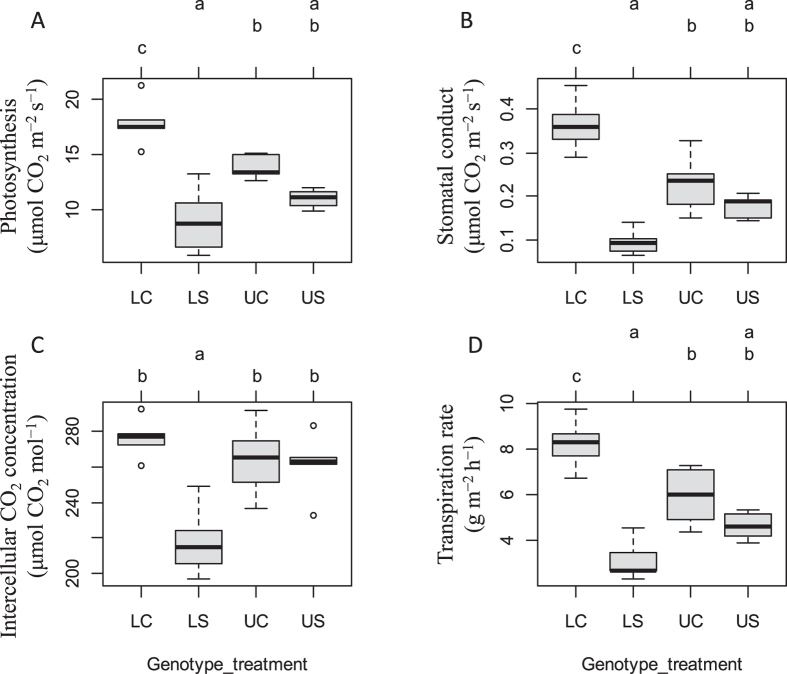
Changes in the photosynthetic machinery in leaves of lowland and upland rice under control and drought stress conditions. (**A**) Rates of photosynthesis in leaves of lowland and upland rice under control and drought stress conditions. (**B**) Stomatal conductance in leaves of lowland and upland rice under control and drought stress conditions. (**C**) Intercellular CO_2_ concentration in leaves of lowland and upland rice under control and drought stress conditions. (**D**) Transpiration rate in leaves of lowland and upland rice under control and drought stress conditions. LC-Lowland rice under control condition; LS-Lowland rice under stress condition; UC-Upland rice under control condition; US-Upland rice under stress condition (Multiple compare test of mean for the four cases was used as a statistical test and the 0.05 statistical significant threshold was chosen.).

GO and KEGG analysis revealed that genes related to photosynthetic machinery were profoundly enriched in orthologs set with Ka/Ks value bigger than 1.5, suggesting that the photosynthesis genes are under strong positive selection during the domestication of upland and lowland rice cultivars. Furthermore, the collocation of genes under positive selection with QTLs related to photosynthesis parameters confirmed that photosystem plays an important role in upland rice for adaptation to drought. To validate and further explore the involvement of the photosynthetic apparatus in drought stress adaptation in rice, we measured various physiological photosynthetic parameters *viz*. photosynthesis, stomatal conductance, intercellular CO_2_ concentration and transpiration rate. In line with the published results for plant under drought stress, our results indicate that photosynthetic activity in leaves are overall down-regulated under drought stress (Fig. 8). Among of them, photosynthesis under stress was 45% lower than under well water conditions in lowland rice varieties. The decreased photosynthesis was accompanied by reduced stomatal conductance, intercellular CO_2_ concentration and transpiration rate which was 73%, 24% and 60% lower under stress compared to well water growth condition. In contrast, less profound impact on photosynthetic machinery by drought stress was observed in the upland rice varieties and the above physiological parameters under drought stress were 21%, 23%, 1% and 22% lower than that under control condition (Fig. 8). These results demonstrated that the relatively higher photosynthetic capacity was maintained under drought conditions in upland rice than in lowland rice, which is probably an important part of mechanisms for upland rice to cope with drought environment.

## Discussion

### Cell death and defense response related genes expressed differentially

The genome-wide expression profiling of rice under drought stress has been investigated previously by using microarray, SSH technique, and most recently by RNA sequencing^1^,^2^,^3^,^4^. In the present report genome-wide expression profiling of two genotypes, IRAT109 and Zhenshan97, which show contrasting drought stress phenotypes was performed at the seedling stage by RNA seq in an attempt to identify the genotype-specific gene expression patterns. The DEGs were enriched mainly in the biological processes containing cell death, apoptosis, defense response and molecular function categories including purine nucleotide binding, ATP binding, protein serine/threonine kinase activity and protein tyrosine kinase activity (Fig. 1). These results indicate that cell death and defense response related genes play key role for drought response, and sufficient supply of energy is crucial for rice growth under drought stress. Most notably, expression patterns of some genes differed drastically between these two genotypes, where a large number of genes were turned on/off. The genes turned off only in the upland or lowland rice might be due to human-induced strong artificial selection for increasing drought stress tolerance in rice. Another plausible explanation for the divergence of gene expression patterns between these two genotypes might be due to the sequence variation in the promoter regions of the corresponding genes^5^. Genes with distinct expression pattern enriched in the stress-responding function categories will be useful sources for further study.

### The sequence variations are important functional markers for the study of molecular mechanisms underlying drought resistance

The availability of genome-wide molecular markers and low-cost genotyping platforms can promote marker-assisted breeding to improve drought tolerance in rice^24^,^25^. Moreover, it has been documented that QTLs for higher grain yield has benefited rice more than in any other cereals, which is mainly due to its continuous cultivation in ecosystems (e.g. upland and lowland)^25^. The importance of molecular markers and QTLs associated with drought tolerance has promoted a number of researches on QTLs mapping related to drought stress with the upland and lowland rice cultivars-derived populations^4^,^5^,^6^,^7^. The functional SNPs and INDELs identified through high-throughput RNA sequencing in the present study largely improve the number of available molecular markers in drought-related research. As these SNPs and INDELs were originated from the transcriptional regions of the genome, the markers themselves, if tightly linked or co-segregated with the target traits most probably, are covered in the potential candidate genes. Furthermore, the conventional and cost-effective gel method was successfully implemented for separating 2bp-INDELs or larger. The present traditional detection of INDELs identified by RNA sequencing would be a specific application which combined the high throughput advantage of novel GBS (genotyping by sequencing) with the easy-going merit of conventional gel detection. Additionally, the distribution of SNPs and INDELs on chromosomes provides the basic clues that people can estimate whether there are enough sequence variations to be used for molecular markers in any specific region to fine mapping QTLs for a specific trait in a separated population derived from these two parents. The high-density SNP and INDEL markers reported here will be a valuable resource for genetic studies and molecular breeding of these two widely used rice genotypes with regards to stress response.

### Photosynthesis pathway related genes experience strong positive selection which might result in drought stress adaptation

In our analysis, about 1041 rice genes showed Ka/Ks value greater than 1.5 and 54 bigger than 2 indicating that relatively abundant genes experience strong positive selection during rice domestication to upland and lowland environments. We propose that strong positive selection pressure was imposed on rice for rapid adaptation following the divergence of ~0.4MYA to the progenitors of the two O. sativa sub-species^26^. Furthermore, we found that the top categories of genes under positive selection were enriched in photosynthesis, translation, regulation of biological process, oxidoreductase activity (supplemental table 4) and even 7 genes with Ka/Ks value higher than 2 encode chlorophyll (Table 2). This is consistent with the KEGG pathway enrichment analysis, which also suggest that a substantial genes under strong positive selection was involved in photosynthesis pathway. Most interestingly, the importance of the photosynthesis related genes were further consolidated as they are collocated with leaf drought-related QTLs, such as net photosynthesis rate, stomata conductance, leaf drying score and so on^7^,^22^. Another few genes under strong positive selection worth to be noted are Os09g28354, Os03g55260 and Os09g15330, encoding a heat shock factor, cytochrome P450 and sugar transporter protein respectively are also collocated with the leaf drought response QTLs. These genes were also consistently reported as abiotic-stress-related genes as non-synonymous SNPs were contained in them, which differentiated the contrasting upland and lowland rice genotypes^27^. Consistently, our physiology results also indicated that upland rice can maintain relatively higher photosynthesis activity under drought conditions than lowland rice. This uniformity between our Ka/Ks data, GO, KEGG annotation, QTLs co-localizations and physiology results and previous study confirm the crucial roles of the photosynthesis related genes under positive selection in drought resistance.

It is not surprising for the importance of the photosynthetic system in rice tolerance to drought stress, instead, we provide transcriptional and genetic confirmation at genome-wide horizon since the critical roles of photosynthesis in plant adaptation to drought has been addressed at multi-scale previously. Basically, the photosynthesis has been suggested as a key physiological process on which drought stress directly impact^28^. Net photosynthesis is primarily limited in drought-stressed G. hirsutum by decreased stomatal conductance along with increases in respiratory and photorespiratory carbon losses^29^. Whereas it appeared that the leaf photosynthesis can be supported ideally by high mesophyll conductance to CO_2_ diffusion gm along with high gm/gs ratio, (gs: stomatal conductance to gas diffusion) and a low Smes/gm ratio (Smes: mesophyll cell surface area exposed to intercellular air space per unit of leaf surface area) in rice while preserving water under drought stress^30^. The alternative electron sinks and cyclic electron flow were also proposed to have key roles in photo protection of PSII and PSI in wheat leaves under drought conditions^31^. A number of genes have been reported to be able to regulate the drought resistance through improving the photosynthetic capacity. CAMTA1 could rapidly change broad spectrum of responsive genes of membrane integrity and photosynthetic machinery for challenging drought stress in Arabidopsis^32^. The over expression of gene LcVDE in Arabidopsis can also increase tolerance to drought stress through increasing the maximum quantum yield of primary photochemistry of PSII^33^. Recently, a bHLH transcription factor PebHLH35 from Populus euphratica has been confirmed to confer drought tolerance through regulating stomatal development and photosynthesis in Arabidopsis^34^. Although the genes encoding proteins for photosynthesis machinery were not detected as differentially expressed at the transcriptional level, our results indicates that the physiological parameters in photosynthesis showed different reducing extent in upland and lowland rice. We proposed that the discriminate performances of photosynthesis in upland and lowland rice under drought stress may be attributed to those photosynthesis-related genes under positive selection between upland and lowland rice. Collectively, the present results and previous studies together consolidate that photosynthesis related genes might contribute profoundly to rice upland adaptation. The positive selected genes identified here, especially those enriched in photosynthetic machinery could be genetically engineered for improving drought tolerance in rice.

In summary, the extensive transcriptome divergence including differential gene expression pattern, strong positive selection between upland rice IRAT109 and lowland rice Zhenshan97 indicates the strong effects of selection during domestication to diverse environments. The strongly positive selected genes located in drought stress related QTLs and enriched in photosynthesis pathway illuminate the critical functions of photosynthetic machinery in plant responding to drought stress. The genes and sequence variations identified in the present study are fruitful resources for the future studies on molecular mechanisms underlying drought resistance and genetic improvement of rice drought tolerance.

## Materials and Methods

### Plant materials, growth conditions and stress treatment

Initially, two rice cultivars namely IRAT109 (*O. sativa* L. ssp. *japonica*) and Zhenshan 97 (*Oryza sativa* L. ssp. *Indica)* were used. IRAT109 is a upland rice variety been frequently used in drought-tolerant breeding programs and QTL mapping studies^7^, whereas Zhenshan 97 is a drought-sensitive cultivar, as the maintainer line for a number of elite hybrids widely cultivated in China. Under water stress conditions, the values of grain yield, tillers per plant and spikelet fertility of upland rice varietie IRAT109 is significantly higher than that of lowland rice mainly due to their excellent performance under drought-stressed conditions i.e. faster rolling of leaves, deeper and thicker root in contrast to lowland rice Zhenshan97^11^. Germinated seeds were sown on PVC pots (9cm diameter and depth) filled with soil under 80% relative humidity in the green house with 14 h/25 °C day and 10 h/15 °C night cycle. Germinating seedlings were kept in well-watered conditions until four weeks. Drought stress was imposed by withholding water supply for one week, which resulted in 7% decrease of soil water content (SWC) in the drought-stressed pots as compared to non-stressed controls and the upland cultivar IRAT109 shows better performance than lowland rice under drought-stressed conditions i.e. faster rolling of leaves. On the seventh day of water stress treatment, leaf tissue of 5 plants from each pot was harvested and pooled together from drought stressed plants of each cultivar, and was immediately stored in −80 °C for further analysis. Two biological replicates were applied for each cultivar.

### RNA preparation, cDNA library construction and Illumina sequencing

Total RNA was extracted from the harvested leaf tissue of each cultivar (IRAT109 and Zhenshan97) by using E.Z.N.A. plant RNA kit (OMEGA Bio-Tek, USA) and following the manufacturer’s protocol. Two biological replicates from each cultivar were used for RNA-sequencing. cDNA libraries were prepared individually for each sample by performing a series of procedures, including poly(A) enrichment, RNA fragmentation, random hexamer-primed cDNA synthesis, linker ligation, size selection and PCR amplification. The complementary DNA (cDNA) libraries for paired-end sequencing were prepared using mRNA-Seq Sample Prep Kit (Illumina) following to the manufacturer’s instructions. The libraries were then sequenced using HiSeq Illumina sequencing platform (Illumina, San Diego, CA). Illumina sequencing was performed at Novogene Bioinformatics Technology Co., Ltd., Beijing, China (www.novogene.cn).

### Read mapping and transcriptome assembly

FastQC analysis was performed to estimate the quality of raw reads (http://www.bioinformatics.babraham.ac.uk/projects/download.html#fastqc). After trimming adapter sequences and filtering low-quality reads with >5% ambiguous bases the clean reads were used to do transcriptome assembly based on the reference genome of *Oryza sativa L.* and updated genome annotation from Ensembl website (ftp://ussd-ftp.illumina.com/Oryza_sativa_japonica/Ensembl/MSU6/Oryza_sativa_japonica_Ensembl_MSU6.tar.gz). The clean reads of each replicate from each cultivar were mapped using TopHat 2.0.11 program with the default parameters set^35^. Zhenshan97 and IRAT109 transcriptomes were reconstructed by cufflinks using the default parameters^36^. Transcriptome assembly generated from the above steps were subsequently merged by Cuffmerge module in Cufflinks package to generate comprehensive transcripts for subsequent gene expression.

### Gene expression analysis

Cuffdiff was used to compare the expression level of transcripts, and to test the statistical significance between two cultivars^36^. To identify most differentially expressed genes, we ranked genes according to their size and sequencing coverage normalized FPKM (fragments per kilo base of exon per million). The log2 fold changes of gene FPKM between two genotypes were tested statistically to determine whether an individual gene expression was altered significantly or not. A false discovery rate (FDR) of 0.01 was used for identifying differentially expressed genes (DEGs). The graph generation was performed using in-house R scripts^37^.

### qRT-PCR confirmation of DEGs

A total of 10 DEGs were randomly selected to verify our RNA sequencing expression data by qRT-PCR. Moreover, to validate the expression pattern of these genes in more cultivars, three upland rice varieties (IRAT 109, Hanmadao 1 and Dandongludao) and five lowland rice cultivars (Zhenshan 97B, IR 24, Minghui 63, Huahuangzhan and Fuhui 838) were grown under the control and drought stress growth conditions same as the plants grown for RNA seq analysis (see above ‘plant materials’ sub-section). Total RNA was extracted from the leaves of each cultivar and treatment by using E.Z.N.A. plant RNA kit (OMEGA Bio-Tek, USA), and three biological replicates were used for qRT-PCR analysis. First-stand cDNA was synthesized by using Superscript III reverse transcriptase (Invitrogen). qRT-PCR was conducted on ABI Prism 7500 real-time PCR system (Applied Biosystems, USA). For qRT-PCR analysis, the gene-specific primers were designed by Primer3 (http://www.ncbi.nlm.nih.gov/tools/primer-blast/) (Additional file 3). The expression level of rice Actin1 gene (accession no. X16280) was used as the internal control with primers forward 5′-TGGCATCTCTCAGCACATTCC-3′ and reverse 5′-TGCACAATGGATGGGTCAGA-3′. q-PCR amplifications were performed in an optical 384-well plate. Each reaction was done in a volume of 25 uL containing 12.5 μL of 2 × SYBR green master reagent (Applied Biosystems, USA), 5.0 μL diluted transcription product and 0.2 μL of each gene-specific primer and 7.1 uL ddH_2_O. The following thermal cycle was used: 95 °C for 3 min and then 45 cycles of 95 °C for 30 s, 60 °C for 30 s and 72 °C for 1 min. Dissociation curve analysis was performed using the following thermal profile: 95 °C for 15 s, 60 °C for 20 s and 95 °C for 15 min. The relative expression levels were determined as described previously^38^.

### Identification and distribution of SNPs and INDELs on genome

To identify the sequence variation between the transcripts of two samples, the BAM files generated from TopHat analysis were used for generating pileup files containing variation information with SAMtools mpileup command^39^. Pileup files created from the SAMtools were viewed with BCFtools to produce a raw VCF file which can be visualized in the command line for the variation information. The following options were selected to filter the SNPs. A minimum read depth of 5, a minimum base phred quality of 30, a minimum mapping quality of 20 were used for efficient SNP and INDEL calling. Based on the SNP information between samples and reference, the SNP information between samples were retrieved using in-house bash scripts. To understand the distribution of SNPs in genes and explore hotspots of SNPs on genomes, the relative distribution of SNPs and genes on chromosomes at a 100 kb sliding windows were examined using in-house Perl script. Distribution pattern was graphed with R script.

### Experimental verification of INDELs markers

To verify the sequence variations identified after bioinformatics analysis, we performed lab experiments. Genomic DNA was extracted from the young leaves of IRAT 109 and Zhenshan 97, and from the individuals of RIL (Recombinant Inbred Line) population using the modified CTAB method^40^ PCR amplification was done with a 20 μL reaction mixture containing 100 ng DNA, 1 x PCR buffer, 100uM dNTPs, 250 μM primers, and 1.2 unit Taq polymerase enzyme. 7% denaturing polyacrylamide gels electrophoresis (PAGE) was used for resolving the PCR products, followed by silver staining^41^. A few representative INDELs are shown in the Fig. 4 between parents and among RIL individuals and the primers used for their PCR amplification are enlisted in the Additional File 4 (Yellow section shows primers used in Fig. 4).

### Ka/Ks calculation

To obtain the transcriptome sequences for each genotype, Trinity, release on 2014_04_13 was run using the clean reads of the two genotypes with the parameters seqType fq, CPU 8, JM 80G^42^. To detect the selection pressure on genes, we estimated the proportion of nonsynonymous to synonymous substitutions (Ka/Ks). The transcript sequences from upland and lowland rice were aligned with the reciprocal BLAST (BLASTN) hit with an e-value of 1e^−20^. Two sequences were defined as orthologous if each of them was the best hit of the other and if the sequences were aligned over 300bp. Using available rice protein database in TIGR, the pairs of orthologous transcript sequences from upland and lowland rice were aligned with the reciprocal BLAST (BLASTX) if the aligned regions were greater than100 amino acids and a hit with the expected e-value was less than 1e^−15^. If no same best match was found in this step, the pair of sequences were discarded. The final transcripts of upland and lowland orthologous from the above steps were obtained using PAL2NAL with the help of corresponding protein sequences^43^. The sequences out of PAL2NAL were used as input file for the calculation of Ka/Ks by applying KaKs_Calculator with MA method (Model Averaging on a set of candidate models)^44^.

### Gene function annotation

To inspect the functions of DEGs and positively selected genes, gene ontology (GO) based enrichment tests were conducted using Singular Enrichment Analysis (SEA) in AgriGO toolkit (http://bioinfo.cau.edu.cn/agriGO/analysis.php)^45^. We selected *Oryza sativa* as a supported species, and all annotated rice genes as background for the GO test of significantly differentially expressed genes. However, for genes with Ka/Ks ratios more than 1.5, 12626 orthologous genes undergoing Ka/Ks analysis were chosen as customized reference for GO enrichment tests. The Fisher statistical test and Yekutieli (FDR under dependency) multi-test adjustment methods were applied, and significant GO terms with p < 0.05 were retrieved. All the corresponding transcripts were also used in searches against the significant threshold e-value ≤ 10^−5 ^46. The enriched pathways were downloaded from the KEGG database and corresponding genes belonging to the pathways and under positive selection were listed in the results.

### Photosynthesis measurement

Five upland rice cultivars IRAT109, Hanmadao1, Hanmadao2, Dandongludao, Taidongludao, and five lowland rice varieties Zhenshan97, IR24, Minghui63, Huahuangzhan, Fuhui838, were grown under the same conditions as the plants used for RNA sequencing (see above ‘plant materials’ sub-section). Net photosynthesis rate, stomatal conductance, intracellular CO_2_ concentration and transpiration rate were measured using a portable photosynthesis system (LI-6400, LI-COR Inc., Lincoln, NE, USA), on the mature part of the fifth leaf. All these parameters were measured at noon inside the growth room for three or four plants for each cultivars. The CO_2_ concentration and temperature in leaf chamber were kept at 400 μmol/mol^–1^ and 25 ± 0.5 °C respectively. Measurements were conducted at saturated photon flux density (1500 μmol m^−2^ s^−1^) by a red-blue light-emitting diode (LED) light source (LI-6400-02B LED; LI-COR).

## Additional Information

**How to cite this article**: Zhang, Z. *et al*. Comparative transcriptome analysis highlights the crucial roles of photosynthetic system in drought stress adaptation in upland rice. *Sci. Rep.* **6**, 19349; doi: 10.1038/srep19349 (2016).

## Supplementary Material

Supplementary Information

Supplementary Dataset 1

Supplementary Dataset 2

Supplementary Dataset 3

## Figures and Tables

**Table 1:** The variations information between the investigated Zhenshan97 and IRAT109 and between them and reference genome.

variation	Counts of variations
Raw variations	166,538
Variations meeting the quality filtering criteria	110,826
Variations between the two investigated samples	77,647
Homologous Variation sites in each sample	72,840
SNPs	70,264
INDELs	2,576
INDELs with over 2 bases variation	811
INDELs with one base vaiation	1765

**Table 2:** Statistics of genes with Ka/Ks > 1.5 and colocated with the drought associated QTLs.

Gene ids	Start(kb)	Ka/Ks	GO terms	Domain	Function annotation	QTLs	Position(kb)	Marker	Trait
Os01g39780	22436	2.014	Biological process	Transmembrane	Located in plastid	qQy_FS_MQM_1	Chr1_23325	RM9	Quantum yield of PSII
Os01g64960	37696	2.299	Abiotic stimulus	Transmembrane	chlorophyll a-b binding	qqP_FW_MQM_2	Chr1_37603	RM1198	Proportion of open PSII
Os04g59440	35337	2.064	Abiotic stimulus	Transmembrane	chlorophyll a-b binding	qMeo_GS_MQM_1	Chr4_34136	RM2799	F’v /F’m
Os07g31830	18911	1.898	Biological process	C2 membrane targeting	GTPase activating protein	qA_FW_MQM_1	Chr7_18959	RM432	Net photosynthesis rate
Os09g24540	14610	2.034	Metabolic process	Rotamase	Peptitidyl-prolyl isomerase	qA_GS_MQM_2	Chr9_14367	RM5657	Net photosynthesis rate
Os09g28310	17189	1.793	Abiotic stimulus	Bzip_1	bZIP transcription factor	qA_FW_MQM_3	Chr9_17643	RM410	Net photosynthesis rate
Os09g28354	17221	1.534	Abiotic stimulus	HSF_DNA binding	Heat shock factor	qMeo_FS_MQM_2	Chr9_17643	RM410	F’v /F’m
Os09g29130	17703	1.784	Nucleotide metabolic	ZF-HD_dimer	ZF-HD proteindimerisation	qA_FS_MQM_2	Chr9_17643	RM410	Net photosynthesis rate
Os09g29490	17932	1.697	Response to stress	Transmembrane	Peroxidase	qGs_FS_MQM_2	Chr9_17643	RM410	Stomatal conductance
Os03g54000	30959	1.711	Cellular homeostasis	Oligopeptide transporter	Oligopeptide transporter				
Os03g54980	31256	1.929	Biological process	Transmembrane	protein family UPF0139				
Os03g55070	31316	1.696	Oxidoreductase activity	UDPG_MGDP_dh	Glucose 6-dehydrogenase	QLds3b	Chr3_30912–31658	RM520-RM293	Leaf drying score
Os03g55150	31391	1.524	Translation factor	eIF-5a	Translation initiation factor				
Os03g55260	31456	1.580	Oxygen binding	Transmembrane	Cytochrome P450				
Os09g15330	93663	1.725	Transporter activity	Suger transporter	Transporter family protein				
Os09g15835	96635	1.544	unknown	DUF220	OBP32pep	QDlr9	Ch9_7888–10784	RM219-RM296	No. of days to leaf rolling
Os09g17152	105230	1.860	unknown	F-box domain	F-box domain containing				

F’v /F’m represents maximum efficiency of open PSII in the light. The top 9 rows show the QTLs reported in reference^22^ and the rest of rows show the QTLs identified from reference^7^.
