# Supplementary material for: Comparative transcriptome analysis highlights the crucial roles of photosynthetic system in drought stress adaptation in upland rice
Source: Sci Rep. 2016 Jan 18;6:19349. doi: 10.1038/srep19349 (PMC4726002; doi:10.1038/srep19349)
Supplement: Supplementary Information [file srep19349-s1.doc]

Comparative transcriptome analysis highlights the crucial roles of photosynthetic system in drought stress adaptation in upland rice

Running title: Drought stress adaptation in upland rice

Zhengfeng Zhang^1^ Yuanyuan Li^2^ Benze Xiao^2*^

1. Hubei Key Laboratory of Genetic Regulation and Integrative Biology, School of Life Sciences, Central China Normal University, Wuhan 430079, People’s Republic of China

2. College of Plant Science and Technology, Huazhong Agricultural University, Wuhan 430070, People’s Republic of China

Zhengfeng Zhang Email: [zhengfeng@mail.ccnu.edu.cn](mailto:zhengfeng@mail.ccnu.edu.cn)

Yuanyuan Li Email: [1178879730@qq.com](mailto:1178879730@qq.com)

Benze Xiao Emal: [benzexiao@mail.hzau.edu.cn](mailto:benzexiao@mail.hzau.edu.cn)

* Corresponding author.

Supplemental Table1 The statistics of transcriptome assembly of IARI109 and Zhenshan97

| **Source** | | **Number of Transcripts** |
| --- | --- | --- |
| **Annotated in Gene Model** | | **68682** |
| **Zhenshan97** | known | 68665 |
|  | novel | 28253 |
|  | total | 96918 |
| **IRAT109** | known | 68665 |
|  | novel | 28783 |
|  | total | 97448 |
| **Merged_samples** | known | 68658 |
|  | novel | 34832 |
|  | total | 103490 |

**Supplemental Table 2 Expressed value and annotation of genes enriched in the significant GO terms**

| **Gene id** | **Zhenshan97_FPKM** | **IRAT109_FPKM** | **Functional annotation** |
| --- | --- | --- | --- |
| Os01g06790 | 7.9276 | 0 | disease resistance protein, putative |
| Os01g41750 | 2.58837 | 0 | expressed protein |
| Os11g45980 | 5.65608 | 0 | NBS-LRR type disease resistance protein, putative |
| Os11g42770 | 8.29771 | 0 | Leucine Rich Repeat family protein |
| Os12g09240 | 1.24935 | 0 | NBS-LRR disease resistance protein, putative |
| Os12g27980 | 1.34098 | 0 | transposon protein, putative, unclassified |
| Os02g02680 | 2.84154 | 0 | receptor-like protein kinase 5 precursor, putative |
| Os04g30660 | 15.141 | 0 | NBS type disease resistance protein, putative |
| Os06g07370 | 8.04776 | 0 | transposon protein, putative, unclassified |
| Os06g22460 | 1.79612 | 0 | disease resistance protein RPM1, putative |
| Os06g44800 | 1.22236 | 0 | transposon protein, putative, Ac/Ds sub-class |
| Os07g17850 | 6.9465 | 0 | transposon protein, putative, unclassified |
| Os08g43000 | 2.99363 | 0 | CC-NBS-LRR, putative |
| Os08g35310 | 7.21227 | 0 | O-methyltransferase, putative |
| Os02g13510 | 170.722 | 18.8366 | receptor-like protein kinase 5 precursor, putative |
| Os04g12840 | 7.21736 | 0 | Similar to OSIGBa0126J24.7 protein. |
| Os10g29820 | 4.65039 | 0 | snRNP protein, putative |
| Os01g07590 | 8.83605 | 92.2555 | universal stress protein domain containing protein, putative |
| Os01g26280 | 0 | 7.53859 | OsWAK receptor-like protein kinase |
| Os01g26390 | 0 | 1.87871 | DUF26-lh.1 - DUF26 kinases have homology to DUF26 containing loci |
| Os02g16060 | 0 | 10.5737 | RGA-1, putative |
| Os03g53250 | 0 | 7.24577 | expressed protein |
| Os04g12080 | 0 | 1.9618 | TKL_IRAK_DUF26-lc.7 - DUF26 kinases have homology to DUF26 containing loci |
| Os04g21880 | 0 | 41.5519 | expressed protein |
| Os04g40570 | 0 | 2.64347 | ABC transporter, ATP-binding protein, putative |
| Os04g58910 | 0 | 2.25062 | receptor protein kinase TMK1 precursor, putative |
| Os05g36050 | 0 | 2.34366 | serine/threonine-protein kinase, putative |
| Os06g34960 | 0 | 14.5899 | TKL_IRAK_DUF26.1 - DUF26 kinases have homology to DUF26 containing loci |
| Os06g38650 | 0 | 1.40076 | receptor-like protein kinase precursor, putative |
| Os06g38730 | 0 | 2.34888 | receptor-like protein kinase precursor, putative |
| Os07g12710 | 0 | 1.87585 | GIY-YIG catalytic domain containing protein, putative |
| Os07g35750 | 0 | 3.17578 | TKL_IRAK_DUF26-ld.3 - DUF26 kinases have homology to DUF26 containing loci |
| Os07g38640 | 0 | 4.38605 | inactive receptor kinase At2g26730 precursor, putative |
| Os07g43570 | 0 | 2.18507 | TKL_IRAK_DUF26-lc.25 - DUF26 kinases have homology to DUF26 containing loci |
| Os08g07330 | 0 | 19.0438 | RGH1A, putative |
| Os08g33000 | 0 | 9.86288 | expressed protein |
| Os09g18594 | 0 | 45.2423 | protein kinase domain containing protein |
| Os10g04180 | 0 | 4.93412 | NB-ARC domain containing protein |
| Os10g10540 | 0 | 20.8166 | cysteine-rich repeat secretory protein precursor, putative |
| Os10g20120 | 0 | 0.891836 | serine/threonine-protein kinase receptor precursor, putative |
| Os11g11550 | 0 | 2.25905 | NBS-LRR disease resistance protein, putative |
| Os11g11580 | 0 | 5.77614 | NB-ARC domain containing protein |
| Os11g11940 | 0 | 8.92889 | MLA10, putative |
| Os11g11960 | 0 | 5.11359 | disease resistance protein RPM1, putative |
| Os11g36950 | 0 | 38.9965 | T-complex protein 1 subunit theta, putative |
| Os11g45180 | 0 | 0.774021 | NBS-LRR disease resistance protein, putative |
| Os12g10330 | 0 | 2.17984 | NB-ARC domain containing protein |

**Supplementalal Table 3. The information of genes with Ka/Ks value bigger than 2**

| **Chr** | **Locus** | **Start** | **Ka** | **Ks** | **Ka/Ks** | **Annotation** |
| --- | --- | --- | --- | --- | --- | --- |
| Chr1 | LOC_Os01g03330 | 1344557 | 1.28622 | 0.557546 | 2.30693 | BBTI3 |
| Chr1 | LOC_Os01g03380 | 1363516 | 1.25764 | 0.571444 | 2.20082 | BBTI6 |
| Chr1 | LOC_Os01g04380 | 1951928 | 1.27738 | 0.575447 | 2.21981 | hsp20/alpha |
| Chr1 | LOC_Os01g05105 | 2393480 | 1.30182 | 0.576212 | 2.25927 | expressed |
| Chr1 | LOC_Os01g08470 | 4179159 | 1.32665 | 0.576404 | 2.30159 | retrotransposon |
| Chr1 | LOC_Os01g10580 | 5639769 | 1.38475 | 0.466935 | 2.96562 | B-box |
| Chr1 | LOC_Os01g13210 | 7363643 | 1.21825 | 0.501927 | 2.42714 | salt |
| Chr1 | LOC_Os01g19170 | 10830045 | 1.25784 | 0.620839 | 2.02603 | polygalacturonase |
| Chr1 | LOC_Os01g39780 | 22436013 | 1.16491 | 0.578137 | 2.01493 | expressed |
| Chr1 | LOC_Os01g50820 | 29188850 | 1.27027 | 0.591234 | 2.1485 | transporter |
| Chr1 | LOC_Os01g52240 | 30030998 | 1.26625 | 0.623724 | 2.03014 | chlorophyll |
| Chr1 | LOC_Os01g64170 | 37269723 | 1.26755 | 0.620369 | 2.04322 | glycosyl |
| Chr1 | LOC_Os01g64960 | 37696778 | 1.28537 | 0.55905 | 2.2992 | chlorophyll |
| Chr1 | LOC_Os01g71090 | 41141674 | 1.27903 | 0.618337 | 2.0685 | xylanase |
| Chr1 | LOC_Os01g73790 | 42738667 | 1.26753 | 0.617894 | 2.05137 | NAD |
| Chr2 | LOC_Os02g03250 | 1302273 | 1.2434 | 0.563712 | 2.20573 | expressed |
| Chr2 | LOC_Os02g10080 | 5250031 | 1.17467 | 0.554709 | 2.11763 | zinc |
| Chr2 | LOC_Os02g10390 | 5468367 | 1.26092 | 0.599532 | 2.10317 | chlorophyll |
| Chr3 | LOC_Os03g05750 | 2866315 | 1.29788 | 0.538612 | 2.40968 | heavy-metal-associated |
| Chr3 | LOC_Os03g08490 | 4367117 | 1.25805 | 0.611249 | 2.05815 | AP2 |
| Chr3 | LOC_Os03g28190 | 16227657 | 1.27932 | 0.610662 | 2.09497 | dirigent |
| Chr3 | LOC_Os03g46052 | 26029239 | 1.28791 | 0.628791 | 2.04824 | expressed |
| Chr3 | LOC_Os03g47270 | 26746797 | 1.46894 | 0.34566 | 4.24968 | GCRP4 |
| Chr3 | LOC_Os03g48750 | 27779086 | 1.27549 | 0.579927 | 2.1994 | Cupin |
| Chr3 | LOC_Os03g53740 | 30809029 | 1.25201 | 0.60458 | 2.07087 | expressed |
| Chr3 | LOC_Os03g59010 | 33591195 | 1.29703 | 0.602773 | 2.15177 | Cupin |
| Chr4 | LOC_Os04g38410 | 22835896 | 1.29244 | 0.583474 | 2.21508 | chlorophyll |
| Chr4 | LOC_Os04g47990 | 28534300 | 1.30364 | 0.500122 | 2.60665 | dof |
| Chr4 | LOC_Os04g51172 | 30297864 | 1.25446 | 0.56185 | 2.23272 | Disease |
| Chr4 | LOC_Os04g59440 | 35337067 | 1.25037 | 0.605822 | 2.06392 | chlorophyll |
| Chr5 | LOC_Os05g01380 | 230784 | 1.26297 | 0.621581 | 2.03187 | polygalacturonase |
| Chr5 | LOC_Os05g15520 | 8777954 | 1.2409 | 0.60283 | 2.05845 | DNA-binding |
| Chr5 | LOC_Os05g33430 | 19645657 | 1.23734 | 0.612524 | 2.02007 | xyloglucanase |
| Chr5 | LOC_Os05g34770 | 20630042 | 1.22638 | 0.52698 | 2.3272 | cytochrome |
| Chr6 | LOC_Os06g04000 | 1635096 | 1.26219 | 0.597152 | 2.11369 | peptidyl-prolyl |
| Chr6 | LOC_Os06g39060 | 23187192 | 1.26264 | 0.598833 | 2.10849 | glucan |
| Chr6 | LOC_Os06g51220 | 30993254 | 1.21663 | 0.52234 | 2.3292 | HMG1/2 |
| Chr7 | LOC_Os07g37240 | 22316268 | 1.26771 | 0.61719 | 2.054 | chlorophyll |
| Chr7 | LOC_Os07g38960 | 23357683 | 1.28577 | 0.573584 | 2.24164 | chlorophyll |
| Chr7 | LOC_Os07g48390 | 28919577 | 1.30231 | 0.572518 | 2.27471 | proline-rich |
| Chr8 | LOC_Os08g33190 | 20654681 | 1.30276 | 0.49932 | 2.60908 | linker |
| Chr8 | LOC_Os08g41290 | 26086542 | 1.31991 | 0.5864 | 2.25087 | AIR12 |
| Chr9 | LOC_Os09g24540 | 14610699 | 1.1643 | 0.57245 | 2.03389 | peptidyl-prolyl |
| Chr9 | LOC_Os09g37910 | 21856872 | 1.22727 | 0.57493 | 2.13464 | HMG1/2 |
| Chr9 | LOC_Os09g38010 | 21907638 | 1.23502 | 0.560494 | 2.20346 | no |
| Chr10 | LOC_Os10g28120 | 14602523 | 1.26631 | 0.62641 | 2.02153 | glycosyl |
| Chr11 | LOC_Os11g05530 | 2490667 | 1.28328 | 0.60363 | 2.12593 | expressed |
| Chr11 | LOC_Os11g10470 | 5710546 | 1.20389 | 0.541095 | 2.22491 | expressed |
| Chr11 | LOC_Os11g10510 | 5744961 | 1.22228 | 0.47419 | 2.57762 | dehydrogenase |
| Chr11 | LOC_Os11g13880 | 7655567 | 1.15984 | 0.567181 | 2.04492 | expressed |
| Chr11 | LOC_Os11g29910 | 17383893 | 1.28911 | 0.610358 | 2.11205 | plastocyanin-like |
| Chr11 | LOC_Os11g47820 | 28838673 | 1.26719 | 0.620216 | 2.04315 | glucan |
| Chr12 | LOC_Os12g06280 | 2996185 | 1.29661 | 0.55773 | 2.3248 | expressed |

**Supplemental Table 4.** The annotation for genes with Ka/Ks value > 1.5 and involved in the photosynthesis pathways including ko terms in KEGG and encoded proteins

| **Photosystem and electron transport system** |
| --- |
| **Photosystem II (P680 chlorophyll a) [OT]** |
| **Other common subunits** |
| LOC_Os03g21560.1 K08902 psb27; photosystem II Psb27 protein |
| LOC_Os01g31690.2 K02716 psbO; photosystem II oxygen-evolving enhancer protein 1 |
| LOC_Os07g04840.1 K02717 psbP; photosystem II oxygen-evolving enhancer protein 2 |
| **Other subunits in plants** |
| LOC_Os02g36850.1 K08901 psbQ; photosystem II oxygen-evolving enhancer protein 3 |
| LOC_Os07g36080.2 K08901 psbQ; photosystem II oxygen-evolving enhancer protein 3 |
| LOC_Os01g64960.1 K03542 psbS; photosystem II 22kDa protein |
| LOC_Os04g59440.1 K03542 psbS; photosystem II 22kDa protein |
| **Photosystem I (P700 chlorophyll a) [OT]** |
| **Main subunits** |
| LOC_Os08g44680.1 K02692 psaD; photosystem I subunit II |
| **Other common subunits** |
| LOC_Os07g05480.2 K02698 psaK; photosystem I subunit X |
| LOC_Os12g23200.1 K02699 psaL; photosystem I subunit XI |
| **Other subunits in plants** |
| LOC_Os12g08770.1 K02701 psaN; photosystem I subunit PsaN |
| LOC_Os04g33830.1 K14332 psaO; photosystem I subunit PsaO |
| **Photosynthetic electron transport** |
| LOC_Os06g01210.1 K02638 petE; plastocyanin |
| LOC_Os01g64120.1 K02639 petF; ferredoxin |
| **F-type ATPase [OT]** |
| LOC_Os03g52660.1 K02113 ATPF1D, atpH; F-type H+-transporting ATPase subunit delta |
| **Antenna proteins** |
| **Light-harvesting chlorophyll-protein complex (LHC) - Plant, Green alga [OT]** |
| **LHCI** |
| LOC_Os07g38960.1 K08908 LHCA2; light-harvesting complex I chlorophyll a/b binding protein 2 |
| LOC_Os02g10390.3 K08909 LHCA3; light-harvesting complex I chlorophyll a/b binding protein 3 |
| LOC_Os08g33820.1 K08910 LHCA4; light-harvesting complex I chlorophyll a/b binding protein 4 |
| **LHCII** |
| LOC_Os01g52240.1 K08912 LHCB1; light-harvesting complex II chlorophyll a/b binding protein 1 |
| LOC_Os03g39610.1 K08913 LHCB2; light-harvesting complex II chlorophyll a/b binding protein 2 |
| LOC_Os07g37550.1 K08914 LHCB3; light-harvesting complex II chlorophyll a/b binding protein 3 |
| LOC_Os07g37240.1 K08915 LHCB4; light-harvesting complex II chlorophyll a/b binding protein 4 |
| LOC_Os04g38410.1 K08917 LHCB6; light-harvesting complex II chlorophyll a/b binding protein 6 |

Supplemental Figure 1. The K-means graphs of subclusters of differentially expressed genes between lowland (Zhenshan97) and upland rice (IRAT109).
